# Duration of Adjuvant Doublet Chemotherapy (3 or 6 months) in Patients With High-Risk Stage II Colorectal Cancer

**DOI:** 10.1200/JCO.20.01330

**Published:** 2021-01-13

**Authors:** Timothy J. Iveson, Alberto F. Sobrero, Takayuki Yoshino, Ioannis Souglakos, Fang-Shu Ou, Jeffrey P. Meyers, Qian Shi, Axel Grothey, Mark P. Saunders, Roberto Labianca, Takeharu Yamanaka, Ioannis Boukovinas, Niels H. Hollander, Fabio Galli, Kentaro Yamazaki, Vassilis Georgoulias, Rachel Kerr, Eiji Oki, Sara Lonardi, Andrea Harkin, Gerardo Rosati, James Paul

**Affiliations:** ^1^ University of Southampton, Southampton, United Kingdom; ^2^ Ospedale Policlinico San Martino IRCCS, Genova, Italy; ^3^ National Cancer Center Hospital East, Kashiwa, Japan; ^4^ Department of Medical Oncology, University Hospital of Heraklion, Iraklio, Greece; ^5^ Mayo Clinic, Rochester, MN; ^6^ West Cancer Center and Research Institute, Germantown, TN; ^7^ Christie Hospital, Manchester, United Kingdom; ^8^ Cancer Center, Ospedale Papa Giovanni XXIII Bergamo, Bergamo, Italy; ^9^ Department of Biostatistics, Yokohama City University School of Medicine, Kanagawa, Japan; ^10^ Bioclinic Thessaloniki, Thessaloniki, Greece; ^11^Department of Oncology, Zealand University Hospital, Køge, Denmark; ^12^ IRCCS - Istituto di Ricerche Farmacologiche "Mario Negri", Milan, Italy; ^13^ Shizuoka Cancer Center, Shizuoka, Japan; ^14^ Hellenic Oncology Research Group, Athens, Greece; ^15^ University of Oxford, Oxford, United Kingdom; ^16^ Kyushu University, Fukuoka, Japan; ^17^ Veneto Institute of Oncology IRCCS, Padua, Italy; ^18^ University of Glasgow, Institute of Cancer Sciences, Scotland, United Kingdom; ^19^ San Carlo Hospital, Potenza, Italy

## Abstract

As oxaliplatin results in cumulative neurotoxicity, reducing treatment duration without loss of efficacy would benefit patients and healthcare providers.

**PATIENTS AND METHODS:**

Four of the six studies in the International Duration Evaluation of Adjuvant Chemotherapy (IDEA) collaboration included patients with high-risk stage II colon and rectal cancers. Patients were treated (clinician and/or patient choice) with either fluorouracil, leucovorin, and oxaliplatin (FOLFOX) or capecitabine and oxaliplatin (CAPOX) and randomly assigned to receive 3- or 6-month treatment. The primary end point is disease-free survival (DFS), and noninferiority of 3-month treatment was defined as a hazard ratio (HR) of < 1.2- *v* 6-month arm. To detect this with 80% power at a one-sided type one error rate of 0.10, a total of 542 DFS events were required.

**RESULTS:**

3,273 eligible patients were randomly assigned to either 3- or 6-month treatment with 62% receiving CAPOX and 38% FOLFOX. There were 553 DFS events. Five-year DFS was 80.7% and 83.9% for 3-month and 6-month treatment, respectively (HR, 1.17; 80% CI, 1.05 to 1.31; *P* [for noninferiority] .39). This crossed the noninferiority limit of 1.2. As in the IDEA stage III analysis, the duration effect appeared dependent on the chemotherapy regimen although a test of interaction was negative. HR for CAPOX was 1.02 (80% CI, 0.88 to 1.17), and HR for FOLFOX was 1.41 (80% CI, 1.18 to 1.68).

**CONCLUSION:**

Although noninferiority has not been demonstrated in the overall population, the convenience, reduced toxicity, and cost of 3-month adjuvant CAPOX suggest it as a potential option for high-risk stage II colon cancer if oxaliplatin-based chemotherapy is suitable. The relative contribution of the factors used to define high-risk stage II disease needs better understanding.

## INTRODUCTION

The addition of oxaliplatin to fluoropyrimidine has been shown to improve the efficacy of adjuvant chemotherapy treatment for patients with colon cancer.^1-3^ The initial two studies included patients with stage II and stage III colon cancer; in the MOSAIC study, 40% had stage II disease, and in the NSABP CO-07 study, 28% of patients had stage II disease.^1,2^ On the basis of these studies, 6-month adjuvant treatment with fluorouracil, leucovorin, and oxaliplatin (FOLFOX) or capecitabine and oxaliplatin (CAPOX) became the standard of care in this setting for stage III colon cancer. However, it was recognized that oxaliplatin resulted in significant cumulative and long-lasting neurotoxicity, and as a result of this, six randomized studies were launched to investigate whether the duration of adjuvant chemotherapy treatment could be reduced to 3 months to reduce toxicity, but without compromising efficacy. International Duration Evaluation of Adjuvant Chemotherapy (IDEA), an academic collaboration, was formed to prospectively analyze the individual patient data from these six studies to determine if treatment duration could be shortened from 6 to 3 months. The results for stage III colon cancer have been published.^4^

**CONTEXT**

**Key Objective**
Six-month adjuvant chemotherapy with a fluoropyrimidine and oxaliplatin doublet is an option for high-risk stage II colon cancer. The International Duration Evaluation of Adjuvant Chemotherapy (IDEA) collaboration has investigated if 3-month adjuvant chemotherapy treatment can be given for colon cancer without compromising efficacy. We report the results from the four IDEA studies that recruited high-risk stage II patients.
**Knowledge Generated**
Although noninferiority was not demonstrated for the overall study population (5-year disease-free survival of 80.7% and 83.9% for 3-month and 6-month treatments, respectively), the duration effect of adjuvant treatment is chemotherapy regimen dependent, 6-month treatment results in significantly more toxicity, and these are in line with the results seen for stage III disease. We have demonstrated that high-risk stage II colon cancers that are T4 or have two or more risk factors have a worse prognosis.
**Relevance**
Three-month adjuvant capecitabine and oxaliplatin treatment can be considered an option for some patients with high-risk stage II colon cancer.

More recently, the practice of adjuvant chemotherapy treatment for patients with high-risk stage II disease has changed with single-agent fluoropyrimidine often given, and while the hazard ratio (HR) for improvement in disease-free survival (DFS) from the addition of oxaliplatin is similar for stage II and stage III disease,^1^ in high-risk stage II disease, overall survival (OS) was not improved by the addition of oxaliplatin to fluoropyrimidine.^5^ Four of the six studies in the IDEA collaboration included patients with high-risk stage II disease as these studies were conceived before the OS results of MOSAIC were known.

The results of the pooled analysis of the high-risk stage II disease individual patient data from the four studies within the IDEA collaboration are presented here.

## PATIENTS AND METHODS

### Clinical Trials and Patients

IDEA was an academic collaboration of clinicians and statisticians formed in 2006. All participants were involved in the six concurrently running randomized phase III clinical trials investigating the duration of adjuvant chemotherapy treatment. Four of these trials such as Short Course Oncology Treatment (SCOT) (ClinicalTrials.gov identifier: NCT00749450; Current Controlled Trials number: ISRCTN59757862, and EudraCT number: 2007-003957-10), Adjuvant Chemotherapy for Colon Cancer with High Evidence2 (ACHIEVE2) (UMIN Clinical Trials Registry number: UMIN000013036), Three or Six Colon Adjuvant (TOSCA) (OsSC number: 2007-000354-31 and ClinicalTrials.gov identifier: NCT0064660), and Hellenic Oncology Research Group (HORG) (ClinicalTrials.gov identifier: NCT01308086) recruited patients with high-risk stage II colon and rectal cancer, and this analysis is of patients from these four studies. All research Protocols (online only) were approved by the relevant institutional review board or ethics committee, and all patients provided written informed consent. All four trials investigated whether 3-month adjuvant chemotherapy with an oxaliplatin and fluoropyrimidine doublet was noninferior to the then standard duration of 6-month adjuvant chemotherapy treatment. In all four studies, the choice of chemotherapy regimen, either CAPOX or FOLFOX, was not randomized and decided by the treating clinician before random assignment to 3-month or 6-month treatment. High-risk stage II disease was defined as having one or more of the following adverse features: T4 disease, poorly differentiated adenocarcinoma, invasion (vascular, perilymphatic, or perineural), inadequate nodal harvest (defined as < 10 lymph nodes in SCOT and < 12 lymph nodes in TOSCA, HORG, and ACHIEVE2), bowel obstruction, or perforation.^6^ The principles of data pooling from these studies were agreed prospectively in a collaborative charter initially drawn up in 2011.

All studies recruited patients with colon cancer; SCOT also recruited patients with rectal cancer. Details of the individual trials are shown in Table 1. All four trials provided individual patient data to the Independent Statistical Center at the Mayo Clinic Rochester for analysis.

**TABLE 1.**
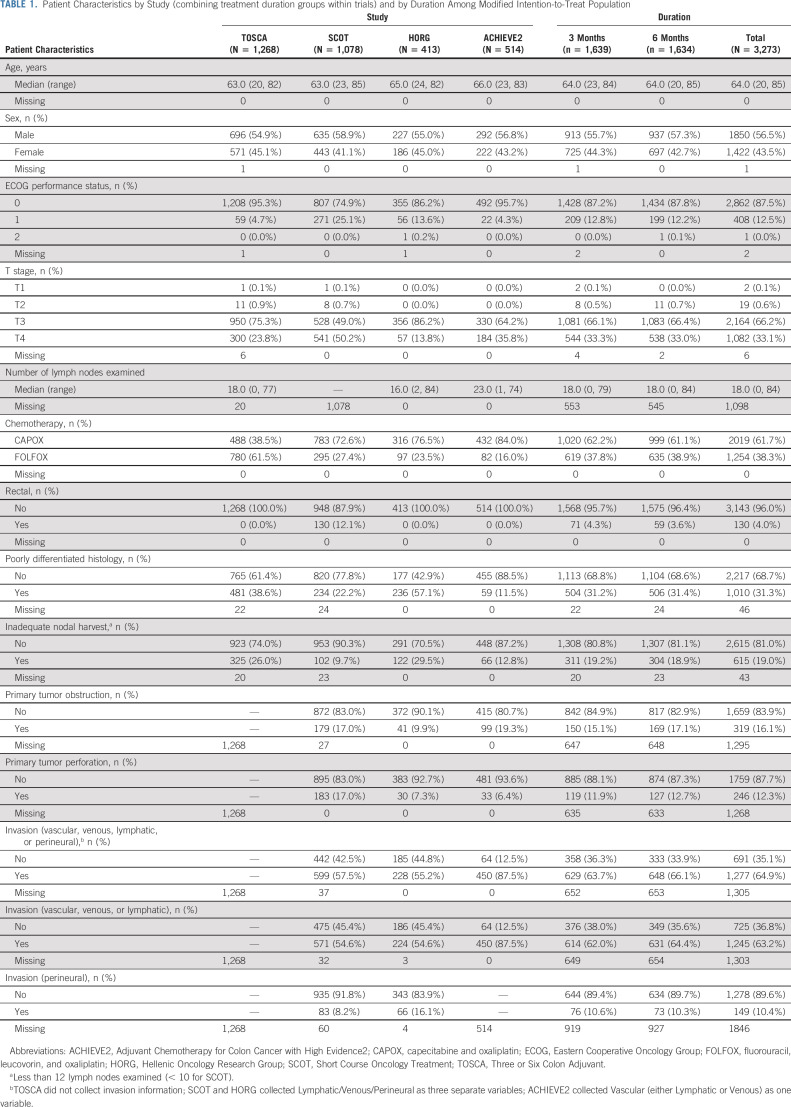
Patient Characteristics by Study (combining treatment duration groups within trials) and by Duration Among Modified Intention-to-Treat Population

### Statistical Design

A final statistical analysis plan was determined prior to the analysis. The primary end point was DFS defined as the time from the date of random assignment to the date of the first recurrence, diagnosis of a second colon cancer, or death from any cause, whichever occurred first. A modified intention-to-treat (mITT) method was used to conduct the primary analysis that included all patients who were randomly assigned and had received at least one dose of chemotherapy; a sensitivity analysis of the primary end point was conducted restricted to the confirmed high-risk stage II patients. Patients were analyzed as per their original random assignment. The DFS HR and associated two-sided CI were estimated by Cox regression analysis stratified by study. The proportional hazards assumption for the stratified Cox model was examined using scaled Schoenfeld residuals. Q statistics and I^2^ values were used to assess the potential heterogeneity of trial-specific DFS HRs comparing 3 and 6 months of therapy. There was preplanned subgroup analysis for the duration effect by regimen (CAPOX *v* FOLFOX), T stage (T4 *v* T1, 2, or 3) disease, poorly differentiated tumors (yes/no), and inadequate nodal harvest. An interaction *P* value < .1 was taken as statistically significant after adjustment using the Benjamini-Hochberg procedure for the tests conducted.

In the MOSAIC study, the addition of oxaliplatin to fluorouracil (FU) improved 5-year DFS from 74.6% to 82.3%^7^ for high-risk stage II. We set the noninferiority boundary HR at 1.2, equivalent to maintaining 60% of the benefit seen in MOSAIC from adding oxaliplatin to FU. This difference corresponds to accepting up to a 3.1% reduction in 5-year DFS (82.3% in 6 m to 79.2% in 3 m FOLFOX or CAPOX). 542 DFS events were required to detect the noninferiority HR of 1.2 for the 3- *v* 6-month arm with 80% power at a one-sided type one error rate of 0.10. The significance level of 0.10 was selected because the number of events required for the typical 0.025 could not be obtained in a reasonable timescale even with a worldwide collaborative effort like IDEA.

## RESULTS

**FIG 1.**
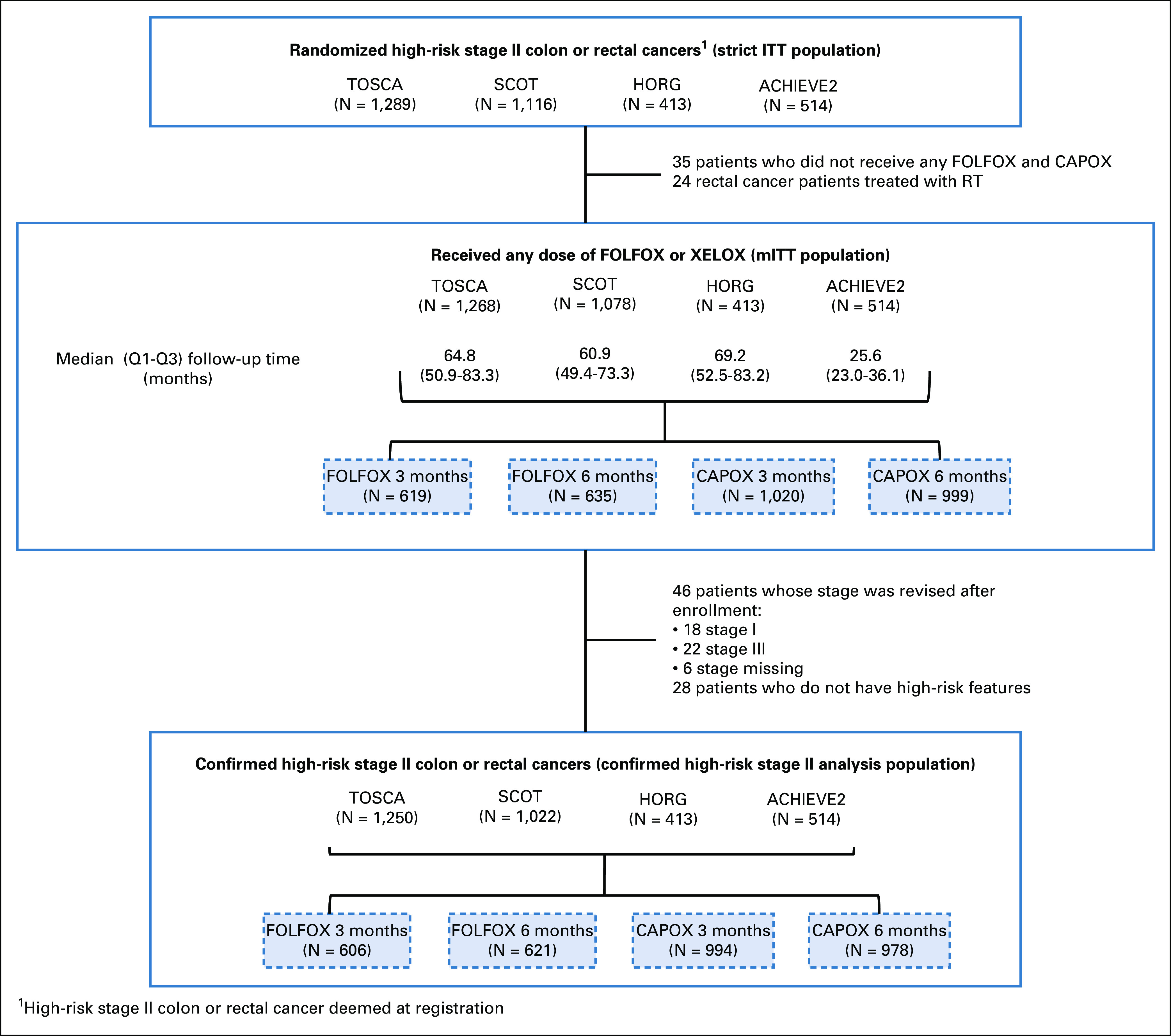
CONSORT diagram. ACHIEVE2, Adjuvant Chemotherapy for Colon Cancer with High Evidence2; CAPOX, capecitabine and oxaliplatin; FOLFOX, fluorouracil, leucovorin, and oxaliplatin; HORG, Hellenic Oncology Research Group; mITT, modified intention-to-treat; RT, radiation therapy; SCOT, Short Course Oncology Treatment; TOSCA, Three or Six Colon Adjuvant.

Between June 20, 2007, and January 31, 2017, a total of 3,332 patients with high-risk stage II colon and rectal cancer were randomly assigned into the four studies. By the time of statistical analysis (December 5, 2018), the median DFS follow-up is 60.2 (59.8-60.5) months. The Consort diagram (Fig 1) shows that 59 patients were excluded from the mITT analysis, 35 because they did not receive any chemotherapy and 24 patients with rectal cancer from SCOT who had received preoperative short-course radiotherapy. There were 3,273 patients in the mITT analysis of whom 62% received CAPOX and 38% FOLFOX.

Patient details and tumor characteristics are presented in Table 1. The percentage of T4 patients in the four studies varied from 14% in HORG to 50% in SCOT. Similarly, patients with poorly differentiated tumors varied from 12% in ACHIEVE2 to 57% in HORG. Microsatellite instability (MSI) data are not available. TOSCA recorded three variables (T4, poorly differentiated tumor, and inadequate nodal harvest), whereas SCOT, ACHIEVE2, and HORG recorded six variables (T4, poorly differentiated tumor, inadequate nodal harvest, obstruction, perforation, and any type of invasion). Patients with one or multiple risk factors from the six risk factors recorded in the SCOT, ACHIEVE2, and HORG studies are shown in the Protocol.

Patient characteristics are well balanced across the randomized arms, and there are no large differences between CAPOX and FOLFOX (Protocol).

### Treatment Intensity

Treatment intensity data are shown in the Protocol. For patients randomly assigned to receive 3-month treatment, approximately 90% received all the planned treatment compared with 65% receiving all the planned treatment in those randomly assigned to receive 6-month treatment (*P* < .0001). The median fluoropyrimidine dose intensity was similar for CAPOX and FOLFOX dropping by approximately 8% from 6-month duration to 3-month duration (*P* < .0001). Median oxaliplatin dose intensity dropped by a greater amount for both FOLFOX (15%) and CAPOX (25%) (*P* < .0001).

### Adverse Events

Adverse events are shown in the Protocol. Overall, patients randomly assigned to 6-month treatment had significantly more adverse events than patients receiving 3-month treatment, especially diarrhea, peripheral neuropathy, hand and foot syndrome, and mucositis. Peripheral neuropathy ≥ grade II was 13% and 36% for those receiving 3-month and 6-month treatments, respectively.

### Efficacy

**FIG 2.**
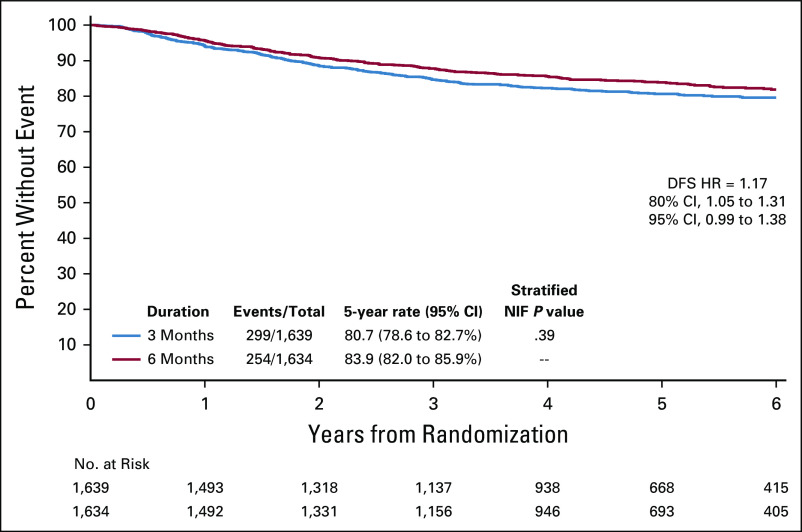
Kaplan-Meier estimates of disease-free survival (DFS) for modified intention-to-treat study population. HR, hazard ratio; NIF, noninferiority.

The Kaplan-Meier plot for DFS in the mITT study population is shown in Figure 2. Of the DFS events, 553 were observed exceeding the 542 required for 80% power. Five-year DFS for those receiving 3-month treatment was 80.7% and 83.9% for those receiving 6-month treatment (HR, 1.17; 80% CI, 1.05 to 1.31). As the CI crossed the noninferiority HR of 1.2, noninferiority for 3-month treatment was not met (*P* = .39). A sensitivity analysis in the confirmed high-risk stage II patients gave virtually identical results.

There was no evidence of nonproportional hazards (*P* > .10 via assessment of Schoenfeld residuals) or of heterogeneity in HRs across individual trials (Q-statistic [*P* value] = 4.24 [.24]; I^2^ [*P* value] = 29.28% [.55]).

**FIG 3.**
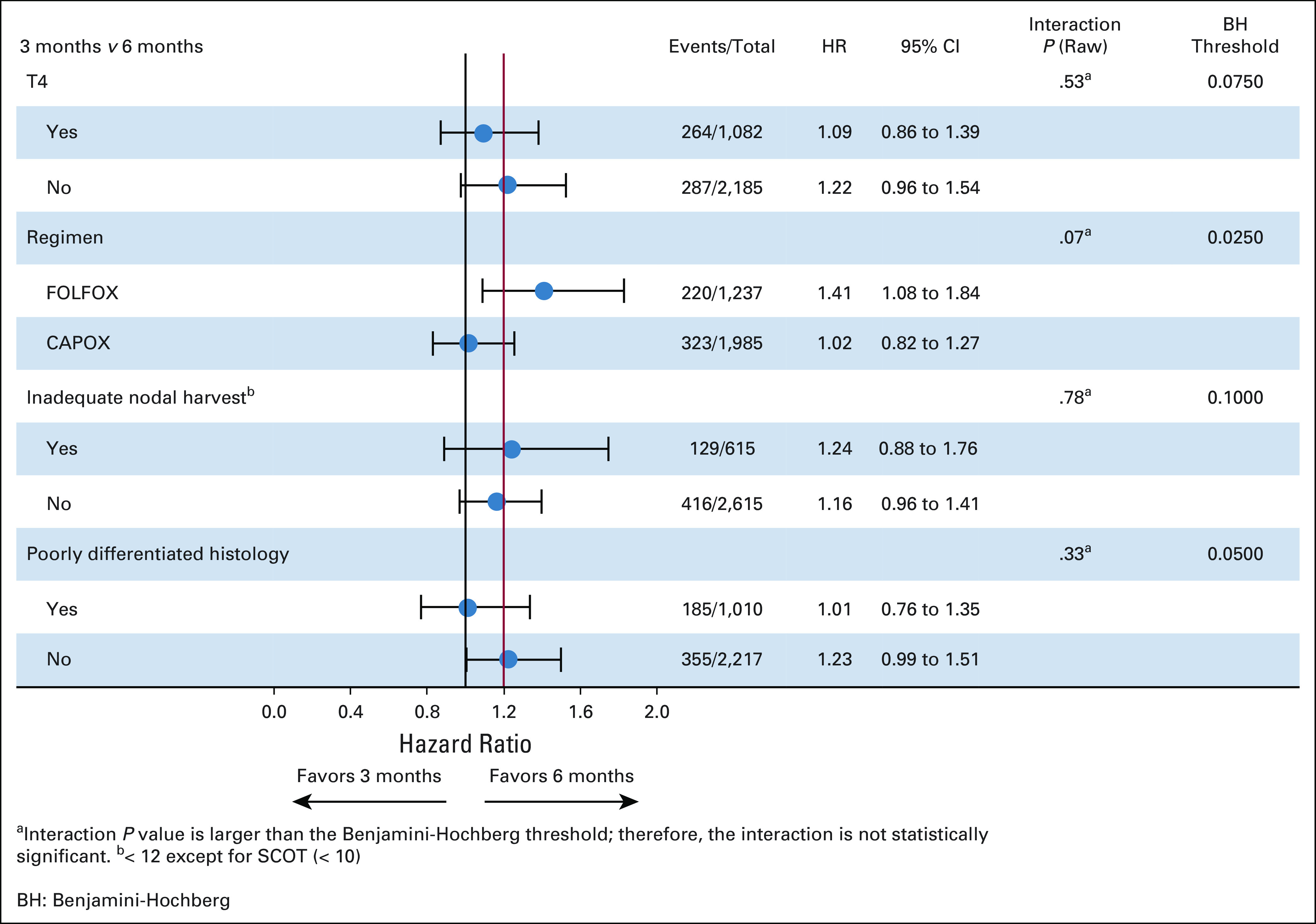
Forest plot for preplanned subgroup analyses. CAPOX, capecitabine and oxaliplatin; FOLFOX, fluorouracil, leucovorin, and oxaliplatin; HR, hazard ratio; SCOT, Short Course Oncology Treatment.

**FIG 4.**
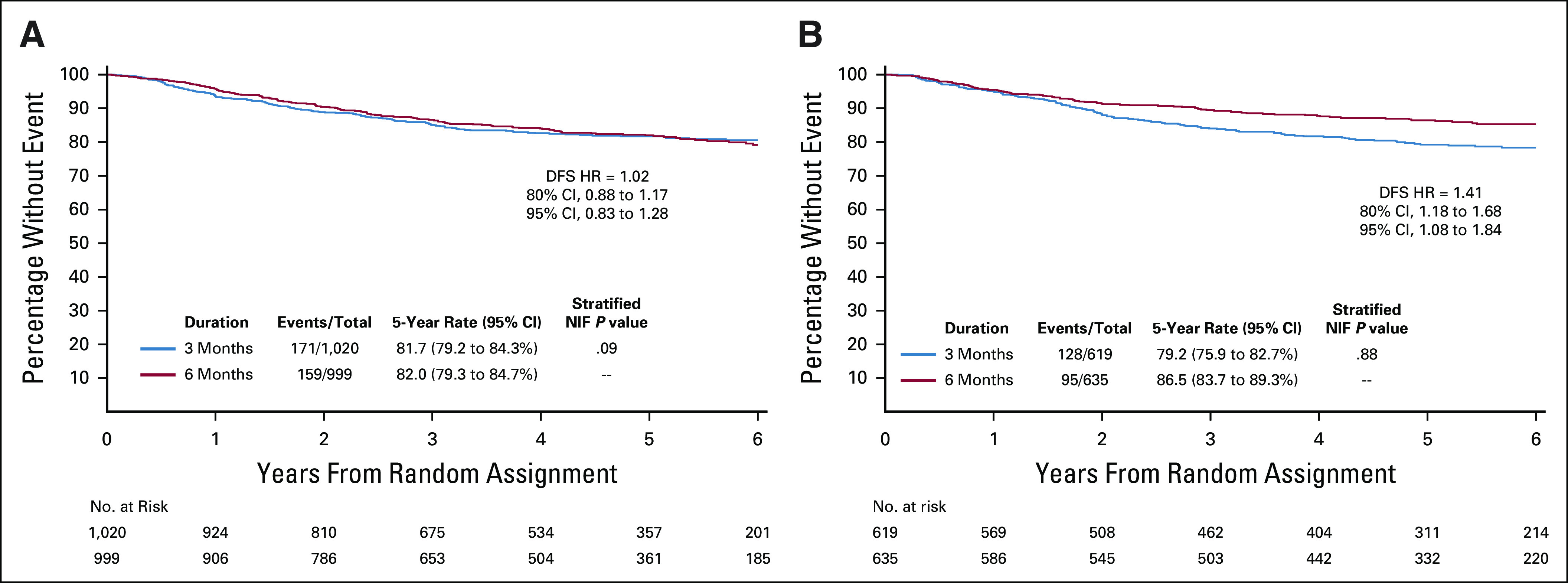
(A) Kaplan-Meier estimates of disease-free survival (DFS) among capecitabine and oxaliplatin (CAPOX)–treated patients and (B) Kaplan-Meier estimates of DFS among fluorouracil, leucovorin, and oxaliplatin (FOLFOX)–treated patients. HR, hazard ratio; NIF, noninferiority.

There were four planned subgroup comparisons: chemotherapy regimen (CAPOX *v* FOLFOX), T-stage (T4 *v* T1-3), poorly differentiated tumor (yes or no), and inadequate nodal harvest. A Forest plot of the HRs in these subgroups is shown in Figure 3. Choice of chemotherapy regimen was the only group that showed a marked difference in the duration effect. The HR for CAPOX was 1.02 (95% CI, 0.82 to 1.27), and the HR for FOLFOX was 1.41 (95% CI, 1.08 to 1.84). We had a preset significant level for interaction of 10%; therefore, the chemotherapy regimen achieved this in the unadjusted analysis, but when the analysis was adjusted for multiple comparisons, this was not the case. In view of the marked observed difference in HRs, the Kaplan-Meier curves for CAPOX and FOLFOX are shown in Figure 4 along with 80% and 95% CIs.

**FIG 5.**
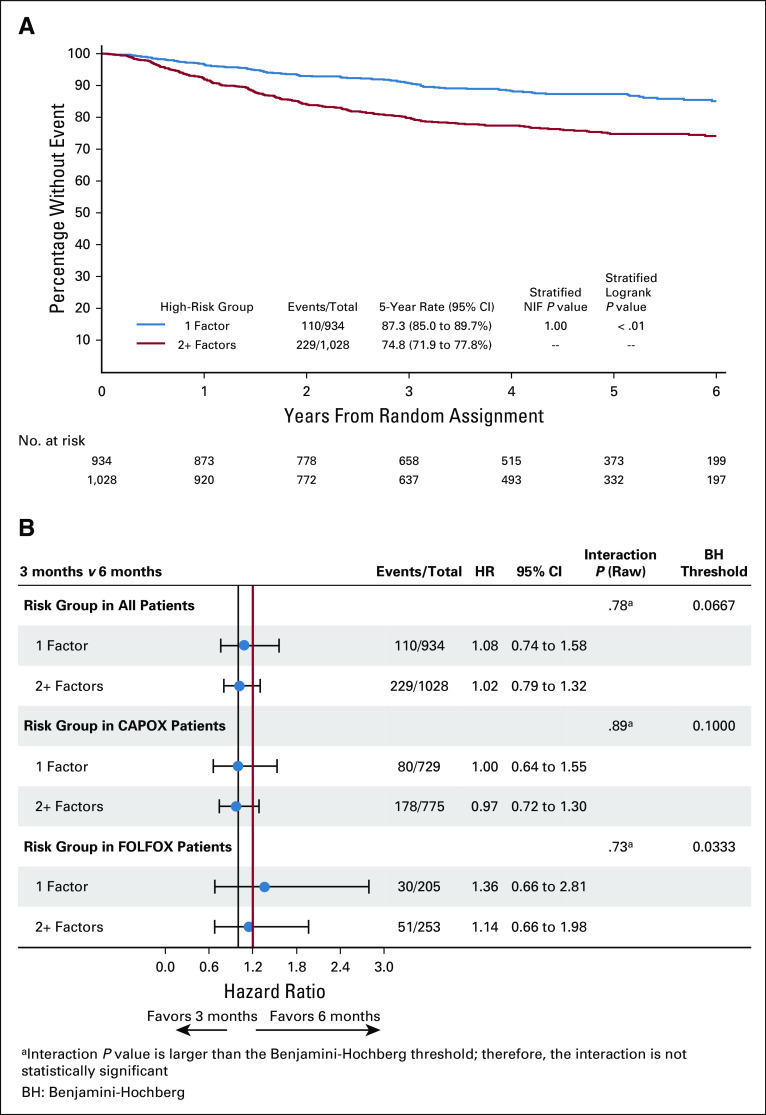
(A) Kaplan-Meier estimates of disease-free survival (DFS) for patients from Short Course Oncology Treatment (SCOT), Adjuvant Chemotherapy for Colon Cancer with High Evidence2, and Hellenic Oncology Research Group comparing those with one risk factor and those with two or more risk factors. (B) Forest plot for patients with one or two or more high-risk factors and chemotherapy regimen received. CAPOX, capecitabine and oxaliplatin; FOLFOX, fluorouracil, leucovorin, and oxaliplatin; HR, hazard ratio; NIF, noninferiority.

**FIG A1.**
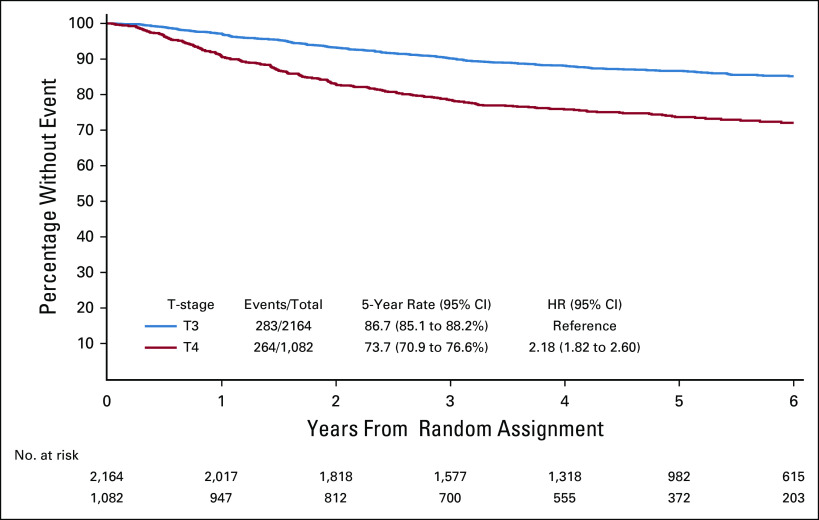
Kaplan-Meier estimates of disease-free survival for patients with T3 disease compared to T4 disease.

An exploratory analysis of the effect of the number of high-risk factors on DFS for the three studies (SCOT, ACHIEVE2, and HORG) that collected data on six high-risk factors was performed comparing those that had just one risk factor and those with two or more risk factors. Kaplan-Meier curves are shown in Figure 5A. Patients with two or more risk factors had a significantly worse DFS (74.8%) than those with just one risk factor (87.3%). The effect of the chemotherapy regimen and the number of risk factors are shown in Figure 5B. We were also able to show from all four studies that high-risk stage II patients with T4 disease have a worse outcome than those with T3 disease (Appendix Fig A1, online only).

## DISCUSSION

Patients with stage II colon cancer represent a heterogeneous group. Stage II patients benefit from adjuvant chemotherapy treatment. The ACCENT adjuvant meta-analysis^8^ looked at 20,898 patients in 18 adjuvant FU-containing trials, 33% of which had stage II disease. This showed an overall improvement in an 8-year survival of 5.4% (from 66.8% to 72.2%). Similarly, the QUASAR study^9^ showed a 3.6% improvement in OS compared with no adjuvant chemotherapy. In recent years, high-risk features have been identified. These include T4 disease, poorly differentiated tumors, bowel obstruction or bowel perforation, < 12 lymph nodes examined, and lympho-vascular and perineural invasion. In stage II disease, only patients with proficient mismatch repair benefit from FU monotherapy in the adjuvant setting.^10^ A limitation to the study is we do not have MSI data as MSI assessment only became standard after completion of accrual to the studies. This will be available in the future for some patients.

Stage II patients with proficient mismatch repair and any risk factors should be considered for chemotherapy treatment, which may include oxaliplatin. When these four studies were conceived, there was evidence that the addition of oxaliplatin improved DFS by 3.8%.^1^ However, more mature data showed no difference in OS for stage II disease.^5^ With high-risk stage II disease, 5-year DFS was increased from 74.6% to 82.3%, and OS at 6 years was increased from 83.3% to 85.0% by the addition of oxaliplatin, but neither were statistically significant.

Although high-risk stage II features have been widely accepted, we do not know their relative impact on prognosis or if any indicate which patients will benefit from adjuvant chemotherapy. There is considerable variation in the percentage of high-risk features between studies. Patients with T4 disease ranged from 14% in the HORG study to 50% in the SCOT study, poorly differentiated tumors from 12% in the ACHIEVE2 study to 57% in the HORG study, and patients with inadequate nodal harvest from 10% in the SCOT study to 30% in the HORG study. The percentage of patients receiving CAPOX varied from 39% in the TOSCA study to 84% in the ACHIEVE2 study.

We did not know if having more than one risk factor resulted in a worse prognosis, but an exploratory analysis using data from three of these studies has shown that patients with two or more risk factors have a significantly worse prognosis than patients with only one risk factor. Only 130 patients had rectal cancers (4% of total), and the applicability of the overall results to this subgroup must be seen in the light of this.

The primary end point of this study showed 80.7% (95% CI, 78.6% to 82.7%) 5-year DFS with 3-month chemotherapy and 83.9% (95% CI, 82.9% to 85.9%) with 6-month treatment, with an absolute difference of 3.2%. The HR was 1.17 (80% CI, 1.05 to 1.3), which crossed the noninferiority margin of 1.2, and so noninferiority was not proven. We do not yet have sufficiently mature OS data as for adjuvant treatment studies in high-risk stage II colon cancer, at least a 6-year follow-up is recommended for an accurate assessment of OS.^11^

There was a preplanned analysis looking at the following variables: chemotherapy regimen, T4 disease (yes or no), poorly differentiated tumor (yes or no), and if there had been an inadequate nodal harvest (yes or no) (Fig 3). Of these four variables, only chemotherapy regimen showed a marked difference. This effect of chemotherapy regimen was similar to that seen in the IDEA collaboration for stage III colon cancer.^4^ Although the *P* value for interaction between regimen and treatment duration was not statistically significant after adjustment for multiple testing, it is worth noting that the 80% CI for CAPOX did not cross the noninferiority margin (Fig 4A), while that for FOLFOX indicates superiority for 6-month treatment.

When the high-risk stage II analysis results are considered alongside the stage III results from the IDEA collaboration, we have data from 16,107 patients, which clearly demonstrates the effect on the duration of adjuvant chemotherapy treatment is different for patients receiving CAPOX compared with those receiving FOLFOX. For CAPOX, 3-month treatment is noninferior to 6-month treatment, but for FOLFOX, chemotherapy of 6-month duration is superior to 3-month treatment. Similarly, in high-risk stage II patients and stage III patients, giving 6-month chemotherapy treatment results in significantly more toxicity. This is especially true for neurotoxicity that has been shown to last for significant periods of time and affect the quality of life.^12,13^

For patients with high-risk stage II disease receiving adjuvant chemotherapy, it is accepted that no OS benefit was shown by the addition of oxaliplatin although an improvement in DFS was seen. We have demonstrated that high-risk stage II patients with two or more risk factors have a worse prognosis and those with T4 disease also have a worse prognosis than those with T3 disease. In view of this worse prognosis, patients with either T4 disease and/or more than two risk factors could be considered for combination treatment. If oxaliplatin-containing adjuvant treatment is recommended in these patients, then the balance between efficacy and toxicity has to be carefully considered. Although noninferiority could not be demonstrated for 3-month treatment in the overall study population, the absolute difference in DFS between 3-month and 6-month CAPOX treatment is only 0.3%, and 3-month treatment results in significantly less toxicity, meaning that balancing efficacy and toxicity 3-month CAPOX can be considered. However, for high-risk stage II disease, adjuvant FOLFOX chemotherapy cannot be recommended as 3-month treatment is inferior to 6-month treatment, and 6-month treatment results in significantly more toxicity, so taking into account, both reduced efficacy and increased toxicity FOLFOX chemotherapy of any duration cannot be recommended. The results of the IDEA collaboration on duration of adjuvant chemotherapy only apply to patients receiving an oxaliplatin and fluoropyrimidine doublet. If patients with high-risk stage II disease receive single-agent fluoropyrimidine treatment such as capecitabine, we have to recommend the current standard duration of 6 months.

It should also be noted that the choice of 3 months of FOLFOX as adequate treatment for low-risk stage III patients based on IDEA^4^ would seem inconsistent with the results reported here for FOLFOX in high-risk stage II. We also note that the trend for better outcome with longer treatment duration for patients with T4 in IDEA is not reflected in these data. However, it should be noted that the prognosis of patients with high-risk stage II cancers can be worse than that for those with low-risk stage III cancers.^14^

Further research to better define the risk factors for high-risk stage II disease and identify those patients who benefit from adjuvant chemotherapy (especially combination chemotherapy) is needed. There is increasing evidence that circulating tumor DNA can identify patients more likely to relapse and hence potentially benefit from adjuvant chemotherapy.^15^ Circulating tumor DNA assessment should be in all future trials and may be helpful in deciding which stage II patients receive chemotherapy.

It should be recognized that the noninferiority conclusion in patients who received 3 months of CAPOX in this analysis was based on a less stringent control of false-positive rate of 10% and subgroup findings. However, regimen-dependent subgroup analyses were prospectively planned, and more importantly, the results are highly consistent with that shown in stage III population. IDEA has previously shown that 3-month treatment with CAPOX has become a standard-of-care adjuvant chemotherapy for patients with stage III disease. Although noninferiority has not been demonstrated in the overall population, the convenience, reduced toxicity, and cost of 3-month adjuvant CAPOX suggest that the standard of care for high-risk stage II colon cancer can be considered to be either 3-month CAPOX (if considered for oxaliplatin-based chemotherapy) or 6-month single-agent fluoropyrimidine.
